# Acknowledgement to the Reviewers

**DOI:** 10.1038/s41439-022-00227-4

**Published:** 2022-12-20

**Authors:** Issei Imoto

**Affiliations:** Director, Aichi Cancer Center Research Institute, Nagoya, Japan

The Editor-in-Chief greatly appreciates the generous support from peer reviewers for the journal, *Human Genome Variation*. The following individuals have reviewed papers submitted from January 2022 to December 2022. We would like to express our gratitude for their generous contribution in 2022.

Yoko Aoki

Siddharth Banka

Brian Chung

Qiang Ding

Atsushi Fujita

Kantaro Fujiwara

Maki Fukami

Long Guo

Kohei Hamanaka

Tomonobu Hasegawa

Shin Hayashi

Naomi Hino-Fukuyo

Kouyuki Hirayasu

Minoru Horie

Masashi Ikeda

Tsuneo Ikenoue

Hidehito Inagaki

Ken Inoue

Kinya Ishikawa

Rodolfo Iuliano

Timothy Jinam

Mitsuhiro Kato

Yosuke Kawai

Minae Kawashima

Jun Kido

Hie Lim Kim

Kengo Kinoshita

Kae Koganebuchi

Tomoki Kosho

Hiroki Kurahashi

Kenji Kurosawa

Charles Larsen

Yoshio Makita

Koichi Matsuda

Hiroyuki Mishima

Fuyuki Miya

Satoko Miyatake

Naoya Morisada

Shinichi Moriwaki

Mitsuko Nakashima

Masahiro Nakatochi

Akira Narita

Nao Nishida

Chikako Nishigori

Emiko Noguchi

Daiju Oba

Soichi Ogishima

Kazutaka Ohi

Masafumi Ohtsubo

Takafumi Ojima

Satomi Okano

Tetsuya Okazaki

Hitoshi Osaka

Yoshihiko Saito

Shinji Saitoh

Yoshiki Sekijima

Kenji Shimizu

Keiko Shimojima Yamamoto

Shigeru Suzuki

Masatoshi Takagi

Chisen Takeuchi

Mariko Taniguchi-Ikeda

Takahito Wada

Rui Yamaguchi

Keiichiro Yamamoto

Ken Yamamoto

Toshiyuki Yamamoto

Akihiro Yasue

Terry-Lynn Young

